# SAMHD1-Dependent and -Independent Functions of HIV-2/SIV Vpx Protein

**DOI:** 10.3389/fmicb.2012.00297

**Published:** 2012-08-10

**Authors:** Mikako Fujita, Masako Nomaguchi, Akio Adachi, Masami Otsuka

**Affiliations:** ^1^Research Institute for Drug Discovery, School of Pharmacy, Kumamoto UniversityKumamoto, Japan; ^2^Department of Microbiology, Institute of Health Biosciences, The University of Tokushima Graduate SchoolTokushima, Japan; ^3^Department of Bioorganic Medicinal Chemistry, Faculty of Life Sciences, Kumamoto UniversityKumamoto, Japan

**Keywords:** Vpx, HIV-2, SIV, SAMHD1, reverse transcription, dNTP, nuclear import, Vpr

## Abstract

Both human immunodeficiency virus (HIV) and simian immunodeficiency virus (SIV) encode a unique set of accessory proteins that enhance viral replication in the host. Two similar accessory proteins, Vpx and Vpr, are encoded by HIV-2. In contrast, HIV-1 encodes Vpr but not Vpx. Recent studies have indicated that Vpx counteracts a particular host restriction factor, thereby facilitating reverse transcription in myeloid cells such as monocyte-derived macrophages and monocyte-derived dendritic cells. This mechanism of counteraction is similar to that of the accessory proteins Vif and Vpu which antagonize other host factors. In 2011, the protein SAMHD1 was identified as the restriction factor counteracted by Vpx. Studies have since revealed that SAMHD1 degrades deoxynucleoside triphosphates (dNTPs), which are components of viral genomic cDNA, in order to deprive viruses of dNTPs. Although interactions between SAMHD1 and Vpx continue to be a major research focus, Vpx has also been shown to have an apparent ability to enhance nuclear import of the viral genome in T lymphocytes. This review summarizes the current knowledge regarding SAMHD1-dependent and -independent functions of Vpx, and discusses possible reasons why HIV-2 encodes both Vpx and Vpr, unlike HIV-1.

## Introduction

Human and simian immunodeficiency viruses (HIV/SIVs) carry a unique set of accessory proteins, Vif, Vpx, Vpr, Vpu, and Nef, which enhance viral replication in the host. Of these accessory proteins, Vpx is unique to HIV-2-type viruses, defined in this paper as the HIV/SIVs carrying both Vpr and Vpx, such as HIV-2, SIVsmm (Sooty mangabey), and SIVmac (Rhesus monkey) (Fujita et al., 2010). Vpr and Vpx are small proteins of approximately 100 amino acids and similar sequence (approximately 20–25% similarity). Both Vpr and Vpx are predicted to have a similar structure consisting of three major helices (Khamsri et al., 2006). In contrast, while HIV-1 carries Vpr, it does not carry Vpx. The answer to the question why HIV-2 viruses encode these two similar proteins while HIV-1 carries only one remains elusive, and must await the determination of their functional details.

Extensive research over the past decade has revealed that lentiviruses carry genes for accessory proteins that overcome host antiviral factors. The first such accessory protein identified was Vif, which inactivates APOBEC3 proteins, cellular cytidine deaminases that restrict the replication of retroviruses by hypermutating viral cDNA and/or inhibiting reverse transcription (Sheehy et al., 2002; Goila-Gaur and Strebel, 2008; Kitamura et al., 2011). Vif reduces the amount of APOBEC3 through proteasome-mediated degradation and other degradation-independent mechanisms. The second major finding in this area was that the viral protein Vpu counteracts host BST-2/tetherin, which normally blocks the release of virions by directly tethering viral particles to the membranes of infected cells (Neil et al., 2008; Van Damme et al., 2008; Arias et al., 2011). The mechanism through which Vpu antagonizes the function of BST-2/tetherin may be proteasome/lysosome degradation or relocalization from the cell surface.

Recently, it was reported that the viral accessory protein Vpx inhibits the host restriction factor SAMHD1 in monocyte-derived macrophages (MDMs) and monocyte-derived dendritic cells (MDDCs) (Hrecka et al., 2011; Laguette et al., 2011), stimulating interest in SAMHD1 and Vpx. In addition to inhibiting SAMHD1 in MDMs and MDDCs, Vpx is also capable of enhancing viral replication in T lymphocytes (Guyader et al., 1989; Kappes et al., 1991; Yu et al., 1991; Akari et al., 1992; Gibbs et al., 1994; Kawamura et al., 1994; Tokunaga et al., 1997; Ueno et al., 2003; Doi et al., 2011). In this review, we summarize current research into SAMHD1-dependent and -independent functions of Vpx and discuss the virological significance of this protein.

## SAMHD1-Dependent Functions of Vpx

Several studies have shown that while wild-type HIV-2-type viruses grow well in MDMs, growth of these Vpx-deletion mutants is completely suppressed, demonstrating that Vpx is essential for viral replication in MDMs (Ueno et al., 2003; Fujita et al., 2008a). It is known that Vpx is packaged in virions and functions in the target cell. Independent work in our laboratory and that of another group revealed that Vpx is critical for reverse transcription of the viral RNA genome in MDMs (Fujita et al., 2008a; Srivastava et al., 2008), correcting the long-held misconception that Vpx contributes to nuclear import of the viral genome but does not play a role in reverse transcription. Furthermore, Vpx was shown to induce proteasome-degradation of an unknown restriction factor to facilitate reverse transcription of the viral genome. It was demonstrated that degradation of the unknown factor involves formation of a Cul4-DDB1-DCAF1 E3 ligase complex (Sharova et al., 2008; Bergamaschi et al., 2009; Kaushik et al., 2009). Considerable effort was subsequently directed toward identification of the unknown factor, and in 2011 SAMHD1 was identified as the MDM host factor from co-immunoprecipitation studies of Vpx expressed in THP-1 cells and in 293T cells (Hrecka et al., 2011; Laguette et al., 2011). SAMHD1 has a tandem sterile alpha motif (SAM) and HD domain with potential phosphohydrolase activity. The SAMHD1 protein was initially identified from MDDCs as a homolog of mouse interferon-γ-induced protein (Li et al., 2000), and is upregulated in response to viral infection (Prehaud et al., 2005; Hartman et al., 2007; Zhao et al., 2008). Furthermore, SAMHD1 is believed to be involved in regulating cellular intrinsic antiviral responses (Rice et al., 2009).

**Figure 1:**
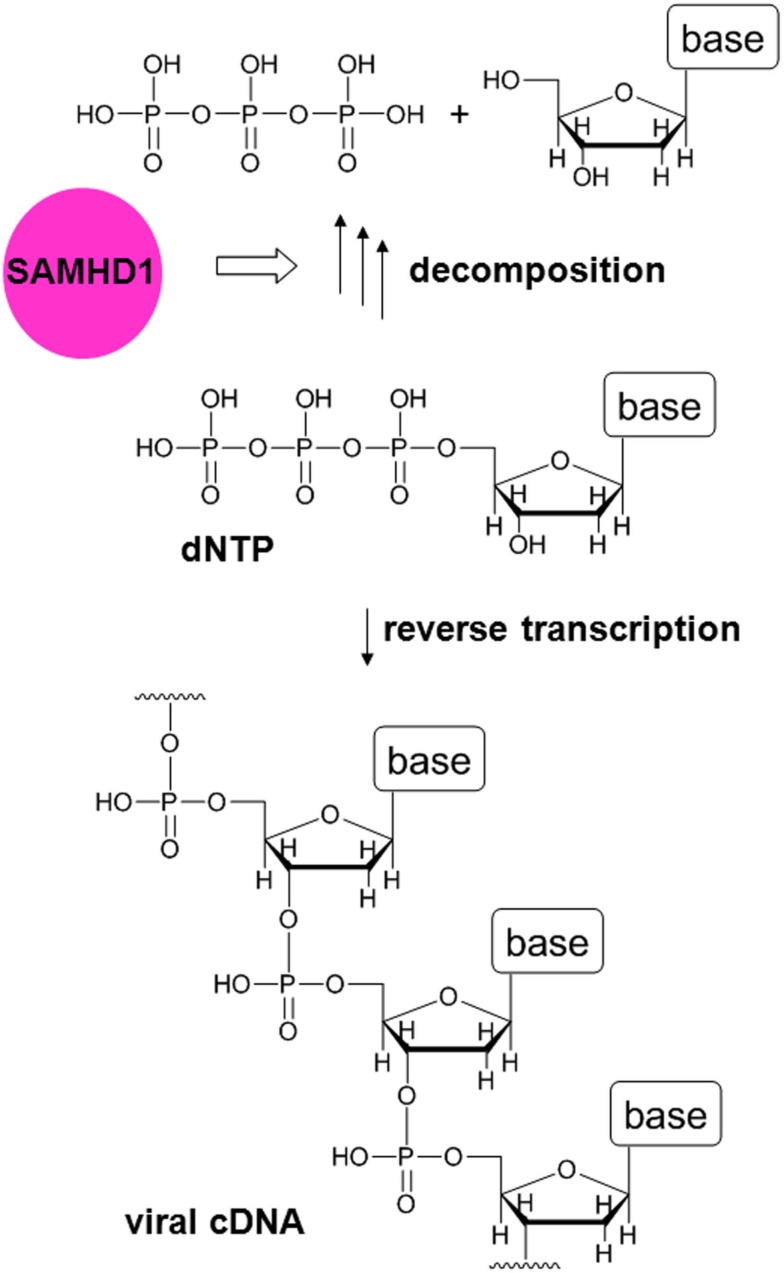
**Function of SAMHD1 in MDMs**. In reverse transcription, viral cDNA is synthesized from dNTPs. The host restriction factor SAMHD1 is a dNTP triphosphohydrolase that degrades dNTPs. Thus, in the presence of SAMHD1, the concentration of dNTPs becomes low and reverse transcription is inhibited.

**Figure 2:**
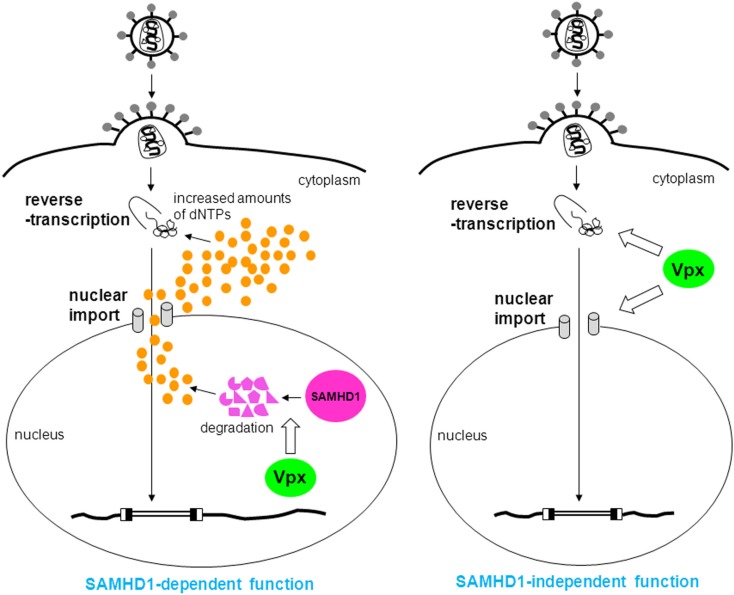
**SAMHD1-dependent and -independent functions of Vpx**. In the former function, Vpx degrades SAMHD1 to increase amounts of dNTPs (orange circles), thus, reverse transcription proceeds (left). In the latter function, Vpx enhances reverse transcription and nuclear import by an unknown mechanism (right).

The identification of SAMHD1 as a target of Vpx was not sufficient to explain all the related phenomena, suggesting the involvement of another factor (Hrecka et al., 2011; Planelles, 2011). However, based on reports indicating that SAMHD1 is a deoxynucleoside triphosphate (dNTP) triphosphohydrolase (Goldstone et al., 2011; Powell et al., 2011), it was hypothesized that SAMHD1 degrades dNTPs (which are small molecule components of viral genomic cDNA) in order to deprive viruses of dNTPs by keeping their concentration low. Lahouassa et al. (2012) recently demonstrated the validity of this hypothesis (Figures 1 and 2). Thus, the additional factor targeted by Vpx appears to be dNTPs. Although dNTPs are utilized for reverse transcription in the cytosol, they are small enough to freely diffuse through nuclear pores in and out of the nucleus. Since SAMHD1 is a nuclear protein (Rice et al., 2009), it is most likely that the concentration of dNTPs in the cytosol is controlled by SAMHD1 in the nucleus. In fact, it was suggested that Vpx-mediated degradation of SAMHD1 is initiated in the nucleus (Brandariz-Nuñez et al., 2012; Figure 2).

In addition to being components of the viral genome, dNTPs are components of the host genome; thus, proliferative CD4^+^ T cells do not express SAMHD1, and maintain the concentration of dNTPs at an optimal level for cell proliferation (2–4 μM; Lahouassa et al., 2012). In contrast, since MDMs do not proliferate, they do not require high levels of dNTPs, and the low dNTP levels (20–40 nM) resulting from SAMHD1-mediated degradation are therefore not harmful to MDMs (Lahouassa et al., 2012). HIV-2-type viruses carry Vpx for proteasome-mediated degradation of SAMHD1 in order to facilitate replication in MDMs. In contrast, HIV-1 does not require Vpx in order to replicate in MDMs (Fujita et al., 2010) because its reverse transcriptase (RT) is capable of catalyzing viral cDNA synthesis from very low levels of dNTPs (Diamond et al., 2004; Lahouassa et al., 2012). The activity of HIV-2 RT is probably lower [Michaelis constant (*K*_m_) of HIV-2 RT is higher] than that of HIV-1 RT, and therefore, to overcome this disadvantage, HIV-2-type viruses may have evolved to carry Vpx.

**Figure 3:**
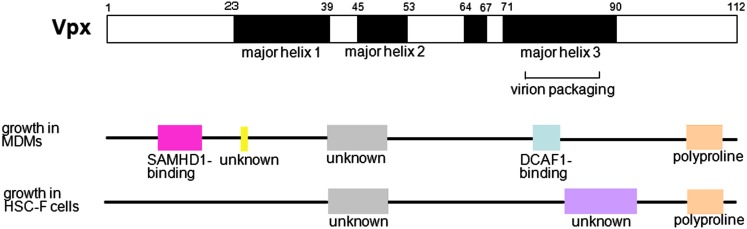
**Mapping of the functional region of Vpx**. In the upper structure, the positions of predicted helices are shown in black (HIV-2 GL-AN clone; Khamsri et al., 2006). The region important for virion packaging is shown. Middle diagram shows regions critical for replication in MDMs: the SAMHD1-binding region (pink), mechanistically unknown amino acid (yellow), mechanistically unknown region (gray), DCAF1-binding region (light blue), and polyproline motif (pale orange). Lower diagram shows regions involved in replication in HSC-F cells: the mechanistically unknown region (gray), mechanistically unknown and unique region (purple), and polyproline motif (pale orange). The polyproline motif is critical for stable expression of Vpx. All functionally important regions, except those for virion packaging, SAMHD1-binding, and DCAF1-binding, are based on our report (Fujita et al., 2008a,b).

We previously mapped the functional region of Vpx involved in viral replication in MDMs (Fujita et al., 2008a,b; Figure 3). It is known that the region in major helix 3 containing amino acids Q^76^ and F^80^ interacts with DCAF1, a subunit of the Cullin4-based E3 ubiquitin ligase complex (Srivastava et al., 2008). This region could overlap with a region that is critical for virion incorporation (Park and Sodroski, 1995; Jin et al., 2001). Gramberg et al. (2010) suggested that another region, which includes amino acids P^9^, N^12^, E^15^, E^16^, and T^17^ in the N-terminal loop, binds to a restriction factor; this region was later confirmed to be a SAMHD1-binding region (Ahn et al., 2012).

We also identified several other functional regions in Vpx, including a central region located between major helix 1 and major helix 2, and a polyproline motif in a loop in the C-terminus (Fujita et al., 2008a,b). We revealed that the C-terminal polyproline motif is critical for stable expression of Vpx. Although the function of the central region remains unknown, it has been confirmed that this region is not involved in virion incorporation.

Following the identification of SAMHD1, several investigators showed that although some cells such as undifferentiated THP-1 cells express SAMHD1, both the wild-type HIV-2-type virus and its Vpx mutant infect these cells to an equivalent degree (Hrecka et al., 2011; Planelles, 2011). There are several possible explanations for the similar infectivity of wild-type and Vpx mutant viruses: (1) These cells contain large amounts of dNTPs, and thus, even in the presence of SAMHD1 there are sufficient quantities of dNTPs for viral replication, and (2) SAMHD1 does not function in these cells for some as yet unexplained reason. A plausible explanation may be posttranslational modification (phosphorylation, etc.) of the protein. Further study will be required to uncover the molecular basis for this phenomenon.

Around the time SAMHD1 was identified, it was reported that another host restriction factor, APOBEC3A, inhibits HIV-1 infection of MDMs, and that APOBEC3A is degraded by Vpx (Berger et al., 2010, 2011). In addition, APOBEC3A reportedly decreases the amount of viral cDNA synthesized during reverse transcription. Presumably, degradation of SAMHD1 alone is not sufficient to enable reverse transcription to proceed smoothly, and therefore degradation of APOBEC3A is also required for viral replication in MDMs, suggesting that Vpx functions to counteract the antiviral effects of both APOBEC3A and SAMHD1. A comparative study between APOBEC3A and SAMHD1 must be performed in order to establish each protein’s contribution to restricting lentivirus infection in myeloid cells.

## SAMHD1-Independent Functions of Vpx

Prior to the time that Vpx was found to act on reverse transcription (Fujita et al., 2008a; Srivastava et al., 2008), it was thought that Vpx is critical for nuclear import of the viral genome (Fletcher et al., 1996; Pancio et al., 2000), based on the results of non-quantitative polymerase chain reaction (PCR) studies. This notion was supported by the tendency of Vpx to localize in the nucleus when Vpx is transduced to a cell solely (Pancio et al., 2000; Mahalingam et al., 2001). It has been well established that Vpx is critical for reverse transcription in MDMs, but this does not preclude participation of Vpx in nuclear import in these cells. We previously identified several Vpx mutants that are defective in both reverse transcription and nuclear import (Fujita et al., 2010) in MDMs, which suggests that Vpx also enhances nuclear import in these cells. We hypothesize that this function of Vpx is SAMHD1-independent, since it is plausible that there is no connection between the amount of dNTPs and nuclear import. Further investigations are underway in order to determine if this is indeed the case.

In T cells, such as peripheral blood lymphocytes (PBLs), peripheral blood mononuclear cells (PBMCs), and cultured simian cell lines immortalized by Herpesvirus saimiri such as HSC-F and M1.3S cells, HIV-2-type viruses grow well, but Vpx-deletion mutants exhibit defective replication (Guyader et al., 1989; Kappes et al., 1991; Yu et al., 1991; Akari et al., 1992; Gibbs et al., 1994; Kawamura et al., 1994; Tokunaga et al., 1997; Ueno et al., 2003; Doi et al., 2011). These results indicate that Vpx is also important for viral replication in T cells. Dispensability of Vpx for the infection of T cells has been believed by some researchers (Bergamaschi et al., 2009; Belshan et al., 2012), but this belief was probably based on the results of infectious experiments using high-titer virus. Our research showed that Vpx enhances nuclear import of the viral genome in HSC-F cells, and that the smaller effect of Vpx on reverse transcription was also observed (Ueno et al., 2003; Fujita et al., 2008a; Figure 2). We mapped the region of Vpx involved in viral replication in HSC-F cells (Fujita et al., 2008b; Figure 3) and found that as is the case in MDMs, the central region and the C-terminal polyproline motif are critical for replication. There is also a unique functional region spanning from major helix 3 to the C-terminal loop, but how this region influences infectivity is unclear. The apparent SAMHD1- and DCAF-1-binding regions are not necessary for viral replication in HSC-F cells, in contrast to MDMs. Furthermore, in HSC-F and M1.3S cells, expression of SAMHD1 was below the detectable level (Nomaguchi, M. and Adachi, A., in preparation). Thus, in these cells, Vpx enhances reverse transcription and nuclear import of the viral genome through an unknown SAMHD1-independent mechanism. Not only cultured cell lines, but also primary T cells are considered to have SAMHD1-independent functions, since SAMHD1- and DCAF-1-binding regions are dispensable for viral replication in PBLs (Fujita, M. and Adachi, A., unpublished data).

It has been reported that Vpx is important for SIV infection in monkeys, and the predominantly infected cells are the intraepithelial T lymphocytes rather than myeloid cells such as macrophages (Hirsch et al., 1998; Belshan et al., 2012). The Vpx in T cells is considered to play a significant role in infection by HIV-2-group viruses *in vivo*. Thus, we strongly suggest that SAMHD1-independent functions of Vpx are also important, although almost all the recent Vpx research has focused on SAMHD1-dependent functions.

## Why Do HIV-2 Viruses have Two Similar Proteins?

Lim et al. (2012) recently revealed that Vpr, a Vpx-related protein found in HIV-1 and HIV-2-type viruses, is not involved in degradation of SAMHD1. Instead, the Vpr carried by HIV-1 and HIV-2 arrests cells in the G_2_ phase of the cell cycle, a function not associated with Vpx (Fletcher et al., 1996; Stivahtis et al., 1997; Fujita et al., 2010; Table 1). This G_2_ arrest is known to be induced via formation of a Cul4-DDB1-DCAF1 E3 ligase complex that includes Vpr as an adaptor. Formation of the complex is followed by proteasomal degradation of an unknown cellular target. This pathway is similar to that involving Vpx, which also functions as an adaptor for the Cul4-DDB1-DCAF1 E3 ligase complex to facilitate proteasomal degradation of SAMHD1 (Ahn et al., 2012). Although the virological significance of the Vpr-mediated G_2_ arrest has not been determined, this function is likely to be important since it is broadly conserved among HIV/SIV. Since the activity of HIV-2 RT is lower than that of the enzyme found in HIV-1-type viruses, HIV-2 may require SAMHD1 degradation in order to increase the concentration of dNTPs, in addition to induction of G_2_ arrest.

**Table 1:** **The roles of Vpx and Vpr in HIV-1 and HIV-2-type viruses**.

	HIV-1	HIV-2 type viruses
Reverse transcription at low dNTP concentrations (in MDMs)	Reverse transcriptase (high activity)	Vpx
Induction of G_2_ arrest	Vpr	Vpr
Enhancement of nuclear import	Vpr (?)^a^	Vpx

*^a^Further study is required (see text)*.

Both of these functions, SAMHD1 degradation and G_2_ arrest, are mediated via the Cul4-DDB1-DCAF1 E3 ligase complex. SIVagm (African Green Monkey) is known to have only one Vpr, which induces both the degradation of SAMHD1 (Lim et al., 2012) and G_2_ arrest (Planelles et al., 1996; Stivahtis et al., 1997; Zhu et al., 2001). Lim et al. proposed that in the evolution of HIV/SIVs, neofunctionalization of Vpr to degrade SAMHD1 resulted in the rapid evolution of the SAMHD1 protein, which induced the birth of a similar protein, Vpx (subfunctionalization), to maximize its SAMHD1-targeting capability. Here, we propose another reason why HIV-2 viruses have similar proteins, Vpr and Vpx. It is known that the region of HIV-1 Vpr spanning from major helix 3 to the C-terminal loop (which includes a cluster of basic amino acids) is critical for induction of G_2_ arrest (Di Marzio et al., 1995; Selig et al., 1997; Jacquot et al., 2007). This region corresponds to the mechanistically unknown and unique region of Vpx required for replication in HSC-F cells (Figure 3; Khamsri et al., 2006; Fujita et al., 2008b), but the corresponding region in Vpx does not contain a cluster of basic amino acids in the C-terminal loop. Induction of G_2_ arrest and enhancement of replication in T cells may be incompatible functions for one protein. The presence of both Vpr and Vpx may facilitate G_2_ arrest and enhancement of HIV-2 replication in T cells, but a full explanation as to why HIV-2 has two proteins that are so similar will require further study.

## Conclusion

Recent findings indicating that Vpx mediates the degradation of SAMHD1 are intriguing, and reveal yet another example of a virus with a means to counteract host defense mechanisms. Table 1 summarizes the roles played by Vpx and Vpr in HIV-1 and HIV-2. HIV-2/SIV Vpx negates the effect of the unique host restriction factor SAMHD1 by inducing its degradation, thereby enabling reverse transcription to occur under conditions of low dNTP concentrations. In addition, Vpx enhances reverse transcription and nuclear import of the viral genome in an SAMHD1-independent manner. We are trying to isolate host factor(s) which concern with SAMHD1-independent function. Different regions of the Vpx protein are involved in mediating SAMHD1-dependent and -independent functions. A number of HIV/SIV accessory proteins have multiple functions, including HIV-1 Vpr (Fujita et al., 2010; Sharifi et al., 2012), Vpu (Nomaguchi et al., 2008; Andrew and Strebel, 2010), and Nef (Foster and Garcia, 2008; Laguette et al., 2010). Importance of those two functions of Vpx in the infected individuals should be revealed in the future.

HIV-1 Vpr has a modest effect on replication in MDMs (Fujita et al., 2010), and there have been reports that Vpr enhances nuclear import of the viral genome in these cells (Tsurutani et al., 2000; Agostini et al., 2002). It is possible that HIV-1 Vpr and HIV-2 Vpx function similarly with respect to nuclear import. However, further study is required to elucidate how these proteins impact nuclear import, since the role of HIV-1 Vpr was demonstrated using non-quantitative PCR, and we could not reproduce this result (Fujita et al., 2010).

Expression of Vpx in MDMs results in the degradation of SAMHD1 and a subsequent increase in the concentration of dNTPs, resulting in an increase in the infectivity of HIV-1. To date, studies of Vpx/SAMHD1 have been mainly restricted to HIV-1, even though Vpx is carried by HIV-2-type viruses. Through future studies involving HIV-2-type viruses, we hope to provide a more complete picture of the roles played by Vpx and Vpr.

## Conflict of Interest Statement

The authors declare that the research was conducted in the absence of any commercial or financial relationships that could be construed as a potential conflict of interest.
